# Spark Plasma Sintering Preparation of Tungsten Carbide-Reinforced Iron-Based Composite Materials: Wear Resistance Performance and Mechanism

**DOI:** 10.3390/ma17235856

**Published:** 2024-11-29

**Authors:** Xiaoyi Zeng, Renquan Wang, Ying Liu

**Affiliations:** 1 School of Materials Science & Engineering, Sichuan University, Chengdu 610065, China; cqzengxiaoyihappy@163.com (X.Z.); wangrq2018@scu.edu.cn (R.W.); 2 Sichuan Provincial for Rare Earth & Vanadium-Titanium Based Functional Materials, Sichuan University, No. 24 South Section 1, Yihuan Road, Chengdu 610065, China; 3 Atlastech Additive Manufacturing Joint Laboratory, Sichuan University, Chengdu 610065, China; 4 Key Laboratory of Advanced Special Materials & Technology, Ministry of Education, Chengdu 610065, China

**Keywords:** non-spherical WC, corrosion, stainless steel, plasma sintering

## Abstract

The spark plasma sintering (SPS) process was used to create iron-based composites reinforced with tungsten carbide (WC) particles of various morphologies, and the effect of WC particle morphology on material wear resistance was systematically investigated. The experiment revealed that the addition of non-spherical WC (CTC-A) significantly altered the composites’ friction coefficient, wear morphology, and wear mechanism. As the CTC-A content increased, the composites’ wear rate decreased at first, then increased, and then decreased again. Composites with a CTC-A concentration of 10% had a minimum wear rate of 2.7 × 10^−6^ mm^3^/(N·m) and peaked at 20%. SEM analysis indicated that the wear mechanism gradually changed from initial oxidative wear to abrasive wear as the CTC-A content increased, and the wear morphology transitioned from smooth to rough with the appearance of numerous abrasives and cracks. The study demonstrated that the low content of non-spherical WC particles during sintering significantly increased the hardness of the matrix by forming carbide phases, while a high content led to increased surface roughness, inducing abrasive wear and reducing wear performance. These findings provide a significant theoretical basis and practical guidance for optimizing the design of iron-based composites.

## 1. Introduction

Stainless steel is widely used due to its excellent mechanical properties and corrosion resistance. However, its low hardness and poor wear resistance limit its application in key moving parts [1]. In order to enhance its wear resistance, researchers have attempted to introduce tungsten carbide (WC) particles into iron-based alloys. Previous studies have shown that the addition of WC particles can significantly enhance the wear resistance of iron-based alloys, especially in plasma spraying and spark plasma sintering processes. For example, Detao et al. [2] discovered that Fe_3_W_3_C carbide can effectively improve the hardness and wear resistance of coatings, whereas Jiang et al. [3] indicated that WC particles can inhibit the growth of high-chromium cast iron grains, thereby enhancing wear resistance. However, the shape of WC particles has an important influence on their performance in cemented carbide. There are differences in the distribution, arrangement, and interfacial bonding strength of WC particles of different shapes in the matrix, and these differences in turn affect the wear resistance of cemented carbide. In previous studies, research on the effect of WC particle shape on wear resistance has mainly focused on qualitative description, lacking systematic quantitative analysis and in-depth theoretical discussion. For example, Padhan et al. [4] found that the wear resistance of nanoscale WC is better than that of microscale WC, and Li et al. [5] found that smaller WC particles can refine the microstructure and be uniformly distributed, which greatly improves their wear resistance. Ren et al. [6] showed that spherical WC particles are easily uniformly distributed in the matrix, but this may affect wear resistance due to the lower interfacial bonding strength, while flaky or needle-like WC particles may exhibit better wear resistance due to higher interfacial bond strength, but their distribution and arrangement may not be homogeneous. However, most of these studies failed to deeply reveal the intrinsic mechanism of the influence of WC particle shape on wear resistance. Therefore, in this study, based on previous studies, stainless steel composites reinforced with different shapes of WC particles were prepared by the spark plasma sintering method through a systematic experimental design to investigate the optimal ratio of non-spherical WC in iron-based alloys, and it is of great significance to further explore the effect of WC particle shape on the wear resistance and to try to reveal its intrinsic mechanism and its wear mechanism.

## 2. Experimental Section

### 2.1. Materials

The experiment used FeCrNi atomized powder and spherical WC powder (CTC-S), with angular WC powder (CTC-A) provided by Zigong Hard Alloy Co., Ltd. (Zigong, China). The chemical composition is shown in Table 1.

### 2.2. Sample Preparation

The raw material powders were mixed according to a certain weight ratio, put into the planetary ball mill for wet grinding, and stirred with tungsten carbide grinding balls for 3 h. After the end of ball milling, the stirred slurry was poured out and put into the vacuum furnace for drying, then put into cylindrical graphite sintering special molds (inner diameter 20 mm) and sintered under vacuum by SPS spark plasma sintering equipment. The optimal sintering parameters obtained by orthogonal testing according to the density and hardness of the alloy are as follows: the sintering temperature was 1100 °C, the pressure was 30 Mpa, the vacuum was −50 MPa, the holding time was 10 min, and the heating rate was about 80 °C/min. The ratio of CTC-S and CTC-A was adjusted, the influence of WC morphology on the wear performance of composite materials was studied, and the sample information is presented in Table 2.

### 2.3. Friction and Wear Performance Testing

Friction and wear experiments were carried out on the Bruker friction and wear test machine. The main parameters included a load of 20 N, a sliding velocity of 5 mm/s, a stroke of 5 mm, and a test time of 30 min. The friction pair used a 6 mm diameter Al_2_O_3_ ball, with the friction coefficient being recorded in real-time during the test process.

### 2.4. Testing and Characterization

The sample was analyzed for phase composition using the DX-2700 X-ray diffractometer produced by Dan Dong Haoyuan Instrument Co., Ltd. (Dandong, China). The main parameters were as follows: copper target Kα radiation, X-ray wavelength λ = 0.15460 nm, a tube voltage of 30 kV, a tube current of 30 mA, a scanning speed of 0.06 °/s, and a scanning angle range of 30–100°. The sample surface morphology, element types, and content were characterized using the JSM-7900F thermal field emission scanning electron microscope produced by JEOL Ltd., Tokyo, Japan, equipped with the Oxford Ultim Max 65 energy dispersive spectrometer produced by Oxford Instruments (Abingdon, UK).

## 3. Results and Discussion

### 3.1. Raw Material Analysis

**Figure 1:**
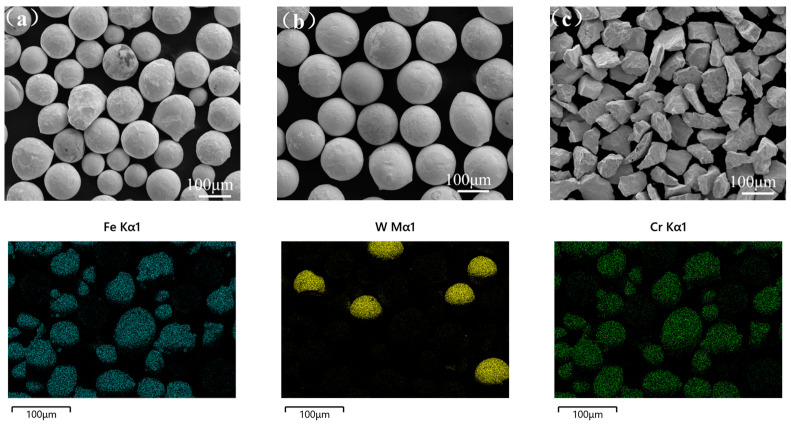
SEM images of raw materials: (**a**) FeCrNi atomized powder; (**b**) spherical WC; (**c**) non-spherical WC.

Using scanning electron microscopy (SEM) to observe the microstructure of raw material powders, Figure 1b,c shows that the powders are mostly spherical or angular particles with a relatively uniform particle size distribution. Subsequently, elemental analysis of the powder after mixing was conducted by energy dispersive spectroscopy (EDS), and Figure 1 shows the analysis results.

### 3.2. Composition

**Figure 2:**
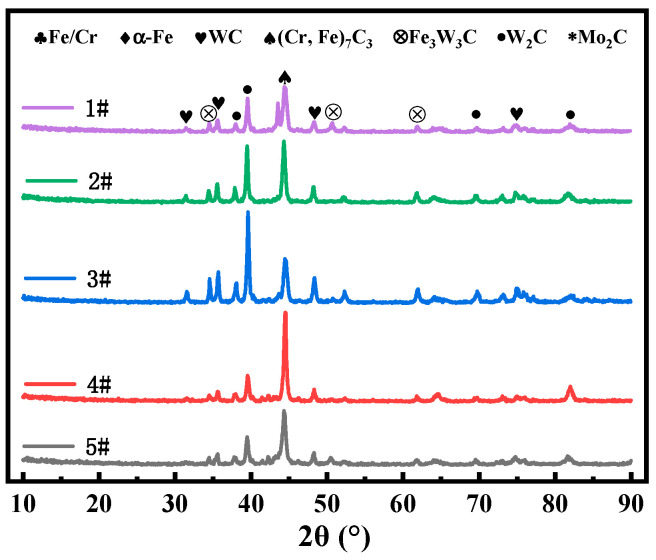
XRD analysis of spherical WC/FeCrNi and non-spherical WC/FeCrNi.

The phase composition of alloys with different contents of CTC-S and CTC-A was analyzed by XRD, as shown in Figure 2. The main diffraction peaks consist of (Cr, Fe)_7_C_3_, W_2_C, WC, and Fe/Cr phases. Secondary carbides such as Fe_3_W_3_C and Mo_2_C were also observed. This is mainly due to the dissolution of WC at high temperatures, where the C element combines with other elements to form secondary carbides [7,8]. The peak value of (Cr, Fe)_7_C_3_ in Sample 2 is the highest, with Sample 1 being the lowest and Sample 3 being the highest when compared to the main peak heights of WC (2θ = 35.7°) and W_2_C (2θ = 39.6°).The differences in height ratios of WC and W_2_C in different samples may be attributed to varying degrees of dissolution of WC during sintering. As the CTC-A content increases, the X-ray diffraction peak of the α-Fe phase shifts towards lower angles. This shift is attributed to the large atomic size of W, which forms a solid solution in the α-Fe matrix, causing lattice expansion. Similar results have been confirmed by Liu et al. [9]. The average grain size of the samples can be calculated using the Scherrer and Wilson equations. [5] Based on this, the full width at half maximum of the diffraction peak of spherical WC is greater than that of non-spherical WC. Therefore, the grain size of non-spherical WC is smaller than that of spherical WC. As a hard phase, WC particles can effectively resist the wear of friction pairs and reduce the volume loss of composite materials. The formation of carbides such as (Cr, Fe)_7_C_3_ also helps to improve the hardness and toughness of the composite material, further enhancing its wear resistance.

### 3.3. Microstructure Analysis

**Figure 3:**
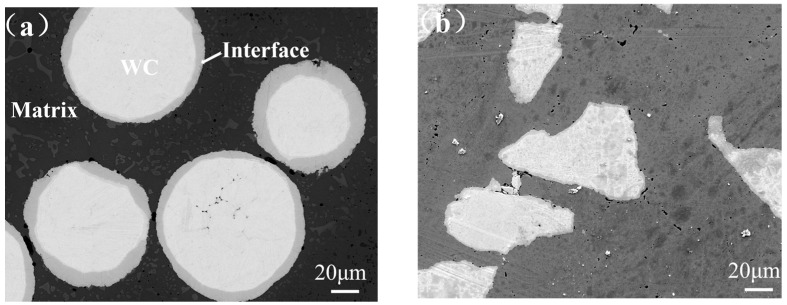
SEM analysis of (**a**) spherical WC/FeCrNi and (**b**) non-spherical WC/FeCrNi.

The phase composition of WC/FeCrNi plasma sintered alloys is essentially the same, but their microstructure and distribution exhibit significant differences due to the different morphologies of WC, as shown in Figure 3. When spherical WC is added, the gray WC maintains an approximately spherical distribution in the black Fe matrix. During solid phase discharge plasma sintering, the sintering neck formed is difficult to diffuse to other powders. The metallurgical reaction generates small black–gray phases, which are rod-shaped with large and tortuous grain boundaries, hindering crack propagation. In contrast, after sintering non-spherical WC the volume fraction of black–gray phases significantly increases, with more pronounced aggregation with the black phase. The morphology of the gray–black original (Cr, Fe)_7_C_3_ phases changes significantly. The rod shape disappears completely, while the size of the gray phase increases and tends to be spherical. It is uniformly distributed on the matrix, and the size of the gray WC phase also slightly increases, with mutual aggregation and diffusion. This indicates that most spherical WC does not melt during sintering, maintaining a relatively intact spherical shape. Non-spherical WC, due to irregular morphology, is more prone to breakage during sintering, resulting in shorter solid–liquid diffusion distance, easier metallurgical reaction, and easier formation of carbide phases after reaction [7]. As the sphericity of WC decreases, the volume fraction of black–gray hard phases continuously increases, transitioning to a more spherical shape, reducing grain boundary area, lowering curvature, and promoting crack propagation. The high melting point of WC acts as a heterogeneous nucleation center for (Cr, Fe)_7_C_3_ and other low-melting-point materials, increasing the sintering nucleation rate, and refining (Cr, Fe)_7_C_3_ grains. Fine-grain strengthening and secondary-phase strengthening work together to improve the mechanical and corrosion resistance of the alloy [8]. Regularly shaped spherical WC provides better thermal contact and more uniform dissolution, whereas non-spherical WC exhibits more significant dissolution at its corners. The irregular morphology and large specific surface area of non-spherical WC particles help to increase the toughness and the number of grain boundaries of the composite, thereby improving its wear resistance.

### 3.4. Analysis of Friction and Wear Performance and Mechanism

#### 3.4.1. The Effect of Adding CTC-A on the Friction Coefficient of the Alloy

**Figure 4:**
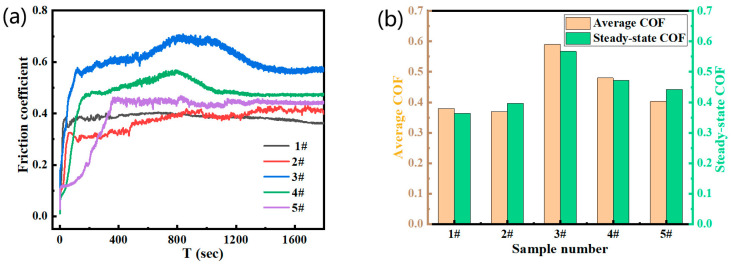
Presenting friction-wear data, showcasing the dynamic friction coefficient curve in (**a**) and the average friction coefficient along with the stable friction coefficient in (**b**).

Figure 4 shows the variation of friction coefficient (COF) during friction experiments of FeCrNi-WC alloys with different contents of CTC-S/CTC-A. In Figure 4a, the COF curves of each sample over time are depicted. It is found that the COF of Sample 1 quickly stabilizes at around 0.36 after a brief running-in period, while Sample 2 stabilizes at 0.39 after 150 s. Sample 3 reaches a peak of 0.69 at 200 s, then fluctuates significantly before finally stabilizing at 0.56. Sample 4 is similar to Sample 3, but the coefficient of friction (COF) stabilizes at 0.47 after 1200 s. Sample 5 stabilizes at 0.43 after a running-in period of 360 s. Overall, samples with different amounts of CTC-A show longer running-in periods and significant fluctuations during the friction process, especially samples 3 and 4. This indicates the instability of alloys containing CTC-A in the formation of the friction layer, possibly due to the formation of (Cr, Fe)_7_C_3_ grains leading to frictional deformation and cracking [9,10].

The average COF and stable COF values of each sample are compared In Figure 4b. The average value of Sample 1 is 0.36, with a stable value of 0.36 as well. Sample 2 has values of 0.39 and 0.41, Sample 3 has values of 0.56 and 0.47, Sample 4 has values of 0.47 and 0.43, and Sample 5 has values of 0.39 and 0.41. This further confirms the analysis above that the increase in CTC-A content will result in COF fluctuations, with a particularly significant impact on stability. Experiments show that adding CTC-A in moderation can improve hardness, but excessive amounts will cause dramatic fluctuations and instability in the friction coefficient, indicating that the friction and wear process is significantly influenced by the internal structure of the alloy [11,12,13].

Research results [14,15,16] indicate that the friction coefficient (COF) at the interface between the iron alloy and Al_2_O_3_ ball is significantly influenced by temperature and the formation of oxides under sliding dry friction-wear conditions. As the friction coefficient increases, so does the friction force, causing a rise in interface temperature. This, in turn, leads to an increase in the formation of oxides, which ultimately has a buffering effect on the wear of the alloy. Meanwhile, the increase in temperature also induces adhesive wear, causing transfer of the alloy at the interface. In the locally transferred area, the alloy is in contact with air more frequently, intensifying the generation of oxides and leading to more oxides adhering to the interface, causing a slight decrease in COF. According to the Ashby model [17], frictional heat can be calculated using a formula that considers factors such as friction temperature, stress, and sliding speed. This formula reflects the close relationship between the friction coefficient, interface temperature, and the generation of oxides. While the addition of oxides can slow down wear, it can also induce adhesive wear, leading to an increase in alloy transfer and oxide generation. This further affects the change in the friction coefficient, resulting in an unstable friction process. This is in line with the experimental results of the fluctuation of the friction coefficient of different contents of CTC-S/CTC-A alloy samples shown in Figure 4.

#### 3.4.2. The Influence of Adding CTC-A on the Morphology of Alloy Wear Tracks

**Figure 5:**
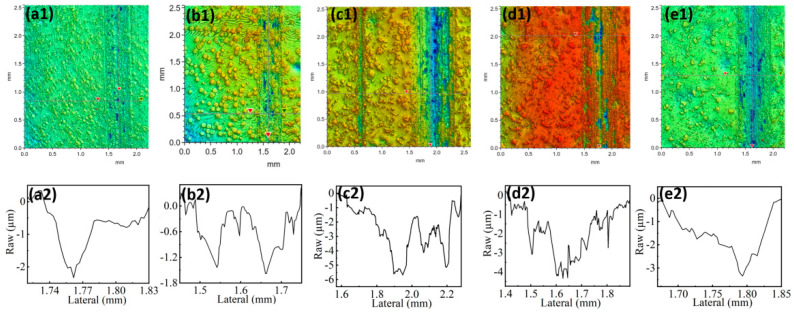
Showing the three-dimensional contour and two-dimensional contour of the central area depth of the wear scar on the surface of the sample after reciprocating friction of the FeCrNi-WC alloy and Al_2_O_3_ balls under dry grinding conditions for 1800 s. The figures labeled (**a1**,**a2**) represent Sample 1, (**b1**,**b2**) represent Sample 2, (**c1**,**c2**) represent Sample 3, (**d1**,**d2**) represent Sample 4, and (**e1**,**e2**) represent Sample 5.

Figure 5a1–e1 depict the optical cross-sectional images of surface grooves on the FeCrNi-WC alloy following dry friction with an Al_2_O_3_ ball. Figure 5a1–e1 illustrate the three-dimensional structure of the sliding friction grooves, showcasing their width and depth, while Figure 5a2–e2 display the two-dimensional morphology of the grooves. With the increase of CTC-A content from 0% to 10%, the groove width on the alloy surface increases from 98 μm to 116 μm, a growth of 16%. Additionally, the groove depth decreases from 2.32 μm to 1.57 μm, a reduction of 48%, as shown in Table 3. However, Sample 2’s alloy has more grooves and fine pits than Sample 1.

As the WC content increases from 10% to 20%, the groove width on Sample 3’s alloy rises from 260 μm to 601 μm, an increase of 81%. The groove depth increases from 1.57 μm to 5.58 μm, a growth of 72%. It can be seen from (c1) that the grooves and fine pits on Sample 3 are more pronounced.

When the CTC-A content increased from 20% to 30%, the scratch width of Sample 4 decreased by 27%, from 601 μm to 475 μm, and the depth decreased from 5.58 μm to 4.32 μm. Despite the decrease in scratch width and depth, the scratch on Sample 4 appeared smoother, with significantly reduced grooves and peak protrusions, indicating better resistance to plastic deformation. Under the same friction conditions, the scratch on Sample 4’s alloy is narrower than that on Sample 3, indicating lower wear and finer, smoother plow furrows and peak protrusions in the scratch. This shows a relatively uniform distribution of the alloy [18,19,20].

Further increasing the content of CTC-A to 40%, the width of the groove of Sample 5 decreased from 475 μm to 195 μm, the depth decreased from 4.32 μm to 3.31 μm, and grooves and depressions reappeared. In the two-dimensional profile of the track, obvious alloy accumulation and up-flow phenomena were observed, reflecting the plastic deformation of the alloy during the friction process. According to the Ashby model, the high temperature generated by friction leads to plastic deformation of the FeCrNi-WC alloy, causing the sides to be squeezed into slopes [21,22]. When compared to Sample 1’s alloy without the addition of CTC-A, the alloy shows improved plastic deformation ability after the addition of CTC-A, thereby reducing its wear rate. This also indicates the significant impact of different contents of WC addition on the wear resistance of the alloy. These observations and analyses suggest that the addition of CTC-A can improve the wear resistance of the alloy, but excessive content may lead to a change in the wear mechanism and affect the overall wear resistance of the alloy.

**Figure 6:**
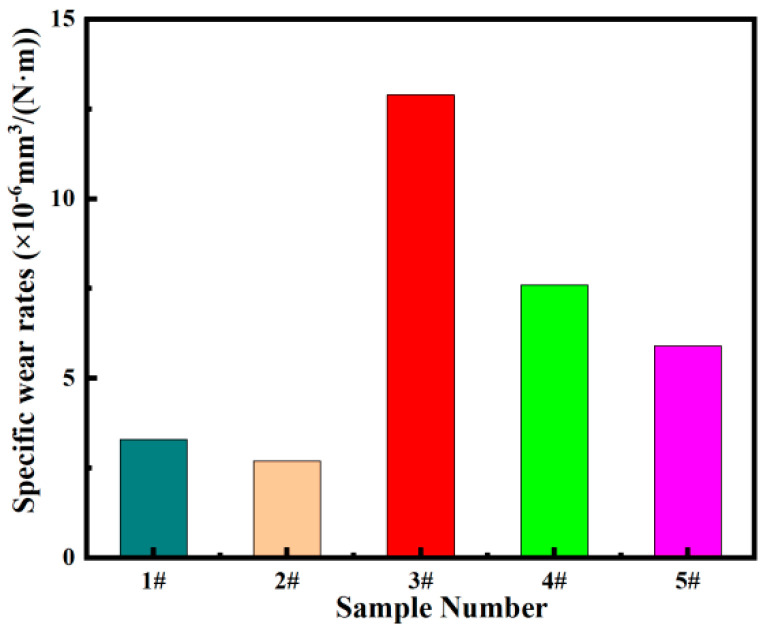
Friction and wear data: volume wear rate.

Figure 6 shows the volume wear rate of the FeCrNi-WC alloy under the conditions of a reciprocating dry sliding wear experiment with 20 N load and an Al_2_O_3_ ball of 6 mm diameter, a sliding distance of 5 mm, a velocity of 5 mm/s, and continuous friction for 30 min. The experiment aims to investigate the effect of different mass fractions of CTC-A on the volume wear rate of the FeCrNi-WC alloy. The data indicates that with the increase of CTC-A content the volume wear rate of the alloy first decreases, then increases, and then decreases again. A sudden change in volume wear rate is observed when the CTC-A content is 20%. Without the addition of CTC-A, the specific wear rate of Sample 1’s alloy is approximately 3.4 × 10^−6^ mm^3^/(N·m). With the addition of 10% CTC-A, the specific wear rate of Sample 2 is about 2.7 × 10^−6^ mm^3^/(N·m); Sample 3 (20% CTC-A) shows a significant increase in specific wear rate to 12.9 × 10^−6^ mm^3^/(N·m); Sample 4 (30% CTC-A) has a specific wear rate of about 7.6 × 10^−6^ mm^3^/(N·m); and Sample 5 (40% CTC-A) has a specific wear rate of approximately 5.9 × 10^−6^ mm^3^/(N·m).This indicates that the addition of CTC-A significantly alters the specific wear rate of the FeCrNi-WC alloy.

Adding CTC-A changed the volume wear rate of the FeCrNi-WC alloy. From the previous analysis, this may be because CTC-A particles increased the surface roughness of the alloy, leading to more severe wear. However, as the CTC-A content continues to increase, the dissolution of angular WC intensifies, enhancing matrix hardness and toughness and effectively resisting wear in the friction pair, resulting in a decrease in volume wear rate. At high temperatures, the friction pair becomes more stable, with alloy surface accumulation and floating phenomena which are plastic deformations caused by frictional force. According to the Ashby model, it suggests that the alloy possesses a specific level of toughness and plasticity that enables it to withstand frictional wear.

In summary, through the study of the friction and wear properties of FeCrNi-WC alloys with different CTC-A contents, it is found that the volume wear rate of the FeCrNi-WC alloy with 10 wt% CTC-A added is relatively low, indicating that this additive ratio is most conducive to reducing wear. This experimental result helps understand the friction behavior and wear mechanisms of FeCrNi-WC alloys, guiding practical alloy design and application to optimize the wear resistance of the alloy.

#### 3.4.3. The Influence of Adding CTC-A on the Micro-Morphology of Alloy Wear Traces

**Figure 7:**
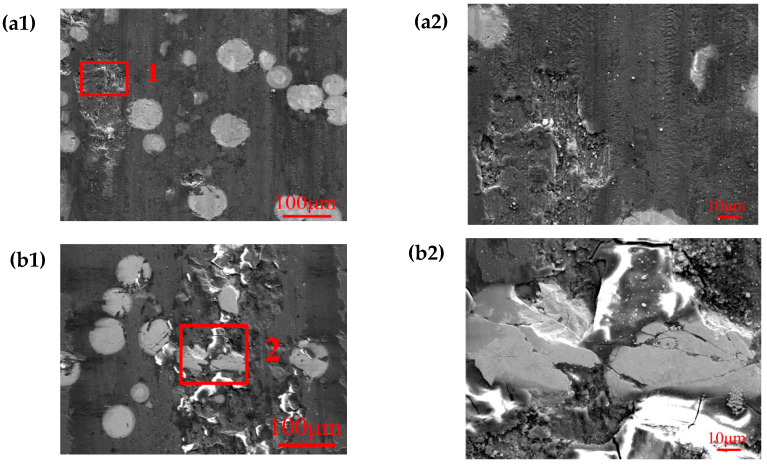

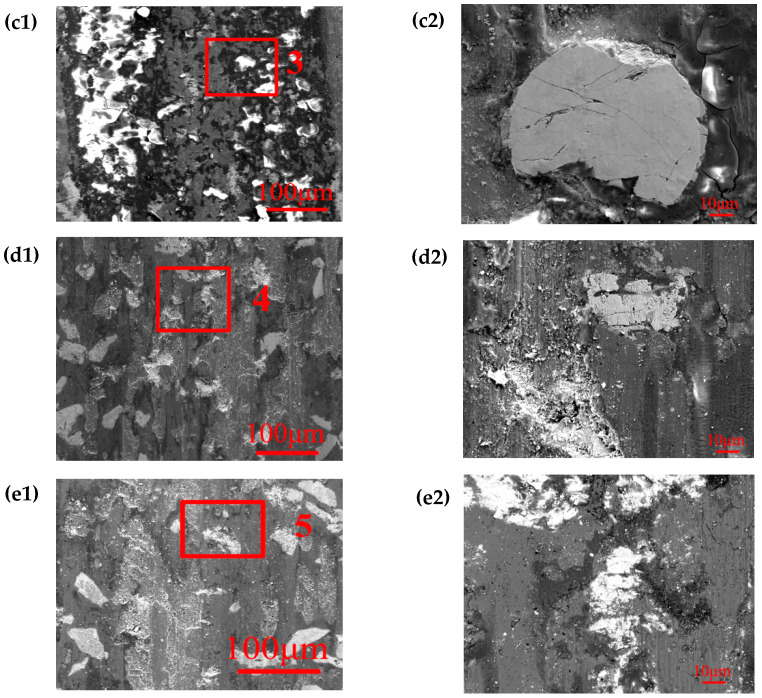
Low (**a1**–**e1**) and high (**a2**–**e2**) magnified surface morphology of worn alloy and morphology of worn debris. (**a1**,**a2**) 1#; (**b1**,**b2**) 2#; (**c1**,**c2**) 3#; (**d1**,**d2**) 4#; (**e1**,**e2**) 5#.

Figure 7a1–e1 show the micro-morphology of the sliding wear scars on the surface of the FeCrNi-WC alloy and Al_2_O_3_ abrasive balls after dry sliding. The transition of friction and wear mechanisms of the alloy is revealed. In Figure 5a1, the surface of the wear scar on Sample 1’s alloy exhibits distinct parallel furrows and patches of oxide combined with the local magnification of the figure can also be clearly seen. Most of these scratches are microplastic deformation abrasion marks, but most of the plow grooves are parallel and relatively smooth, which is the most typical plastic oxidation wear phenomenon of friction wear along the sliding direction. There are a few alloy micro-cracks at the edges of the furrows, indicating brittle fracture occurring under alternating compression and friction. EDS analysis shows that the accumulations at the edges of the furrows are mainly chromium iron oxides, and the ratio of ferric chromium to oxygen atoms is close to 1:1. These chips, because they cannot get rid of the friction surface under the reciprocating continuous rolling of the grinding ball, will be piled up into a layer by the side of the plow groove, which is similar to the stacked laminar structure by the side of the plow groove. However, this laminar structure combines weakly, and under the action of the grinding ball the shear cracks sprout, then the alloy fractures caused by the shedding, as shown in Table 4.

In Figure 7b1, the distribution of oxide on the surface of Sample 2’s alloy shows a wear scar that is discontinuous, with more white oxides and thin, randomly distributed friction plow grooves. However, due to the low content of CTC-A, the alloy structure is not significantly reinforced, resulting in an overall hardness lower than that of the grinding ball, causing the alloy to be worn into an arc shape during the wear process.

Figure 7c1 illustrates that the surface of Sample 3’s alloy scratch is covered by a relatively uniform layer of oxide, with white oxides dispersed on top of this layer. The heightened friction coefficient leads to increased oxidation reactions in the surface material, ultimately resulting in a thicker oxide layer. However, these oxides have poor plasticity, making them prone to fatigue cracks and spalling. EDS analysis indicates that these white oxides are a mixture of alloy oxides and Al_2_O_3_ balls, indicating severe wear of the grinding balls. Despite the isolating, retarding, and lubricating effects of oxides, the mechanical performance differences between oxides and alloys result in crack propagation and fluctuating wear rates.

In Figure 7d1, the surface morphology of Sample 4’s alloy wear scar is flat, but a large amount of white oxide appears. From the magnified image in (d2), black micrometer-level alloy fracture spalling traces can be seen at the lower part of the worn surface, indicating alloy brittle fracture during the formation of plowing furrows in the friction process. In addition, the white oxide in the worn surface area decreases, while it increases in the tearing and spalling areas. These white oxides are uniformly distributed along the sliding friction direction, with a very shallow layered structure. EDS analysis shows that these oxides also contain a mixture of alloy oxides and Al_2_O_3_ spheres. This high-hardness mixture can reduce alloy plastic deformation and suppress plow penetration, but it also increases the hardness of the oxide on the friction contact surface, aggravating the damage to the alloy and Al_2_O_3_ spheres and forming the abrasive source of three-body abrasive wear.

With the further increase in CTC-A addition amount, the accumulation of debris along the furrow edge significantly increases, as shown in Figure 7e1, indicating that more alloy undergoes plastic deformation in the furrow groove, wears off, and adheres to the furrow groove edge. The dense white oxide layer plays a lubricating role, slowing down wear and effectively slowing down the oxidation of alloy in the furrow groove. However, with the increase of CTC-A content, the increase in alloy hardness exacerbates the wear of Al_2_O_3_ balls and abrasive wear. Meanwhile, the shear stress coupling perpendicular to the high temperature and sliding direction causes the soft tissue in the alloy to be displaced, as it is unable to provide sufficient support for hard particles such as CTC-A particles. During the frictional wear process, hard particles such as CTC-A directly protrude and come into contact with the counter-grinding balls, increasing the detachment rate of hard particles. These phenomena reduce the wear resistance of the alloy and exacerbate the wear process.

#### 3.4.4. The Influence of Adding CTC-A on the Wear Mechanism of Alloys

The addition of CTC-A may alter the wear behavior of the alloys, leading to changes in the wear mechanism. By analyzing the effects of CTC-A on the wear properties of alloys, we can gain a better understanding of how this additive impacts the overall performance of the material.

In the alloys we examined, the metal elements Fe and Cr exhibited a strong tendency to react with oxygen. Without the addition of CTC-A, the friction and wear process causes the abrasive ball and alloy surfaces to physically interlock, resulting in concentrated stress on the alloy surface. This leads to the development of cracks and furrows at the defects present on the alloy surface. Under the action of frictional heat, Fe and Cr react with oxygen to form a dense oxide layer which acts as a lubricant and isolates oxygen, reducing friction and wear. However, with the addition of a small amount of CTC-A, more hard phases such as (Cr, Fe)_7_C_3_, and CTC-A will increase the surface roughness, resulting in an increase in the friction coefficient. As the friction force increases, the contact interface temperature rises, further promoting the formation and thickening of the oxide layer. When the oxide layer reaches 1–3 μm, crack propagation leads to the peeling and fracture of the oxide layer due to its brittleness under the action of shear stress [23].

As the content of CTC-A increases to over 30%, friction in the form of microconvexity contact on the surface of the alloy under load induces local stress, leading to brittle fracture of the grinding ball alumina (Al_2_O_3_) and forming abrasives which exacerbate the wear process. After a period of friction, the abrasives gradually wear out, and point contact transitions to surface contact, reducing the cutting effect on the alloy.

**Figure 8:**
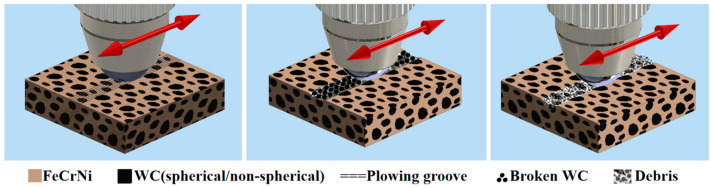
Schematic diagram of the wear mechanism.

Figure 8 shows the schematic diagram of the wear mechanism of the FeCrNi-WC alloy. In the initial stage, the alloy surface contains hard spherical WC particles, resulting in an uneven and non-smooth surface. The primary wear in the initial stage is characterized by oxidative wear, where the material surface reacts with oxygen to form an oxide layer leading to surface wear. As wear progresses, the alloy matrix gradually wears, exposing WC particles. Due to their higher hardness compared to the matrix, these particles preferentially come into contact with the abrasive, forming a friction pair and generating a “protective effect” that effectively mitigates wear development.

During the wear process of the alloy matrix, broken WC particles and matrix fragments are scattered on the surface. Under the influence of high-temperature friction, W and C elements are decomposed and combined with the elements in the matrix to form new carbide phases such as (Cr, Fe)_7_C_3_, significantly increasing the matrix hardness and wear resistance. Subsequently, the wear mechanism gradually transitions from oxidative wear to abrasive wear. This occurs as the abrasives and fragments undergo local plastic deformation, changing the point contact of the grinding ball with the material to surface contact. This, in turn, reduces the cutting pressure on the composite material and weakens the wear.

In conclusion, the wear mechanism of the FeCrNi-WC alloy mainly includes oxidative wear and abrasive wear. Oxidative wear dominates the initial stage, followed by the “protective effect” generated by exposed WC particles, and eventually the wear of grinding balls transition to abrasive wear. This mechanism provides key data and theoretical support for explaining the frictional wear behavior of the WC/FeCrNi-WC alloy, which is beneficial for further optimizing alloy design for practical applications.

## 4. Conclusions

The main conclusions obtained are as follows:

(1) The influence of the coefficient of friction.

The content of CTC-A has a significant effect on the friction coefficient of the FeCrNi-WC alloy, which increases initially and subsequently lowers as the CTC-A content increases. When the CTC-A content is 20%, the friction coefficient reaches the maximum value; when the CTC-A content is 10%, it is the lowest.

(2) Analysis of surface morphology and wear rate.

With the increase of the CTC-A content, the depth and width of the wear groove first grow and subsequently decrease. The wear rate also follows a tendency of lowering, increasing, and then reducing again. When the CTC-A content is at 10%, the volume wear rate is the lowest, at 2.7 × 10^−6^ mm^3^/(N·m).

(3) The microscopic morphology of surface scratches

As the CTC-A content increases, the alloy wear mechanism gradually changes from oxidative wear to abrasive wear. At low contents, it mainly shows oxidative wear, while at high contents a large amount of abrasive wear occurs.

(4) The mechanism of action of WC particles

CTC-A particles can significantly change the wear resistance of the alloy. When the content is minimal, the sintering carbide phase has the potential to dramatically boost the hardness of the matrix. On the other hand, when the content is high, it can cause an increase in surface roughness, leading to abrasive wear and reducing the wear performance.

## Figures and Tables

**Table 1:** Chemical composition of raw material powder (wt%).

Element	C	Fe	Cr	Ni	Si	Mo	B	Mn	W
FeCrNi atomized powder	0.11	74.68	17.15	3.62	1.51	1.27	1.22	0.44	/
Spherical WC (CTC-S)	7.55	/	/	/	/	/	/	/	92.45
Angular WC (CTC-A)	8.86	/	/	/	/	/	/	/	91.14

**Table 2:** Sample ID and sample design (wt%).

Sample Number	CTC-A	CTC-S	Fe-Based Alloy
1#	0	40	60
2#	10	30	60
3#	20	20	60
4#	30	10	60
5#	40	0	60

**Table 3:** 2D abrasion analysis of samples.

Sample	Width (μm)	Percentage (%)	Depth (μm)	Percentage (%)
1#	98		2.32	
2#	116	16	1.57	−48
3#	601	81	5.58	72
4#	475	−27	4.32	−29
5#	195	−144	3.31	−31

**Table 4:** Chemical composition EDS analysis of the alloy friction layer with different CTC-A addition amounts (at. %).

Element	C	O	Al	W	Cr	Fe	Ni	Mo
1#	14.86	32.92	0.14	/	10.99	38.70	1.93	0.45
2#	12.81	38.18	1.63	6.87	6.81	31.99	1.48	0.24
3#	12.81	38.18	5.63	6.87	6.81	27.99	1.48	0.24
4#	13.61	50.05	9.35	5.1	5.06	14.85	0.76	0.21
5#	14.23	47.29	12.54	4.86	4.39	15.72	0.8	0.17

## Data Availability

Data are contained within the article.
